# Lupus nephritis kidney biopsy characteristics and preterm birth

**DOI:** 10.3389/fneph.2024.1402597

**Published:** 2024-07-30

**Authors:** Monica L. Reynolds, Keisha L. Gibson, Tracy A. Manuck, Caroline J. Poulton, Lauren Blazek, Alison M. Stuebe, Susan L. Hogan, Ronald J. Falk, Vimal K. Derebail

**Affiliations:** ^1^ University of North Carolina (UNC) Kidney Center, Division of Nephrology and Hypertension, University of North Carolina at Chapel Hill, Chapel Hill, NC, United States; ^2^ University of North Carolina (UNC) Maternal and Fetal Medicine, Division of Obstetrics and Gynecology, University of North Carolina at Chapel Hill, Chapel Hill, NC, United States

**Keywords:** lupus nephritis, pregnancy, preterm birth, kidney biopsy, family planning

## Abstract

Individuals with lupus nephritis (LN) are at high risk of adverse maternal and fetal outcomes in pregnancy. Outside of pregnancy, proliferative lesions on kidney biopsies are associated with disease progression, but these have not been consistently associated with increased risk in pregnancy. This retrospective, single-center study examines how histologic findings, the timing from kidney biopsy to pregnancy, and the clinical features in the first trimester are associated with preterm birth among individuals with LN. Among 35 deliveries in 31 women, the mean gestational age at delivery was 33.8 weeks. The presence of a urine protein-to-creatinine ratio >0.5 g/g in the first trimester was associated with preterm delivery (81% vs. 36%, *p* = 0.04). Preterm birth was more common in individuals with glomerular crescents on biopsy (89% in those with >20% crescents vs. 50% in those with <20%, *p* = 0.06). A pregnancy occurring within 2 years after a kidney biopsy was more likely to result in preterm birth than if the biopsy was performed more than 2 years prior to conception (82% vs. 23%, *p* = 0.01). The time from diagnostic biopsy may be a surrogate for disease activity, and a 2-year delay from biopsy might allow sufficient time to achieve disease remission. Overall, these data could aid family planning discussions and promote preconception disease optimization for patients and their providers.

## Introduction

Lupus nephritis (LN) in pregnancy is associated with high rates of preterm birth and adverse pregnancy outcomes, including preeclampsia and hypertensive disorders of pregnancy, C-section delivery, and small for gestational age infants (1–4). The preconception clinical risk factors for maternal and fetal morbidity include an elevated serum creatinine, the presence of proteinuria, maternal hypertension, and disease activity (2, 5–8). In a meta-analysis of biopsy-proven LN, both active nephritis and history of nephritis trended toward a significant risk of preterm birth (*p* = 0.08 and *p* = 0.07, respectively) (9). Proliferative LN by histology, defined as class III or IV by the International Society of Nephrology/Renal Pathology Society classification, has not been consistently associated with an increased risk of maternal complications, including preeclampsia (1–3, 8). Outside of pregnancy, a histologic diagnosis of class IV LN and interstitial fibrosis at initial biopsy are associated with progressive disease (10). Knowledge of the relationship of the time at which a diagnostic kidney biopsy is performed and/or between specific histologic features with preterm birth among patients with LN is limited. Further knowledge of these potential associations could aid in preconception counseling and family planning for both patients and their providers. Our goal was to assess kidney histologic findings and timing from kidney biopsy to pregnancy and their association with preterm birth in pregnant individuals with LN.

## Methods

We performed a single-center, retrospective cohort study of individuals with LN enrolled in the Glomerular Disease Collaborative Network (GDCN) registry who delivered at University of North Carolina (UNC) hospitals from 2001 to 2019. The GDCN is a voluntary longitudinal research registry in the southeastern United States that includes data gathered from pathology reports. Among individuals who underwent more than one kidney biopsy, data were utilized from the biopsy closest to delivery. Delivery data came from our institution’s Perinatal Database, which is populated by trained nurse abstractors who review prenatal and peripartum records and enter the medical history, pregnancy, and birth data. Additional data from medical records were abstracted from the Epic@UNC electronic medical record. Preterm birth was defined as <37 weeks’ gestation. Diagnoses of preeclampsia, HELLP (hemolysis, elevated liver enzymes, and low platelets) syndrome, and premature rupture of membranes were based on clinical diagnosis from the discharge summary. Kidney transplant recipients were excluded. Individuals with an initial kidney biopsy postpartum were included if the biopsy occurred within 18 months of delivery. Descriptive statistics such as the percentage, median [interquartile range (IQR)], or mean ± standard deviation described the cohort. Fisher’s exact test assessed the clinical and biopsy characteristics with preterm delivery.

## Results

We identified 35 deliveries among 31 patients with biopsy-proven LN, described in **Table 1**. Preterm birth occurred in nearly two-thirds (63%) of deliveries, and one-third (34%) of all deliveries occurred at <34 weeks’ gestation. The overall mean gestational age at delivery was 33.8 ± 4.6 weeks. The mean gestational age at delivery excluding two twin pregnancies (delivered at 34 and 36 weeks’ gestation) was 33.7 weeks.

**Table 1:** Pregnancies among individuals with lupus nephritis.

Characteristic by delivery %, median, or mean	All deliveries (n=35)	Pre-term birth (63%, n=22)	Full-term birth (37%, n=13)
Maternal race			
African American	54%	55%	54%
Caucasian	23%	23%	23%
Hispanic/Latino	20%	9%	15%
Multigravida	58%	60%	54%
Singleton gestation	94%	91%	100%
Biopsy timing Preconception During pregnancy Postpartum	69% 6% 26%	55% 9% 36%	85% 8% 8%
Primary Biopsy Classification Class II Class III Class IV Class V	3% 23% 49% 26%	5% 18% 59% 18%	0% 31% 31% 38%
First trimester			
Creatinine^*^ (mg/dl) (n=28)	0.63 (0.55-0.89)	0.74 (0.60- 1.01)	0.55 (0.53- 0.62)
UPCR^*^ (mg/g) (n=27)	0.86 (0.20-2.40)	1.3 (0.86- 3.5)	0.32 (0.08- 0.53)
Low C3 (n=26)	50%	50%	50%
Chronic hypertension (n=30)	50%	56%	42%
Hydroxychloroquine use (n=31)	77%	89%	62%
Prednisone use (n=31)	45%	50%	38%
Azathioprine use (n=31)	26%	11%	46%
Calcineurin inhibitor use (n=31)	0%	0%	0%
Third trimester			
Creatinine^*^ (mg/dl) (n=28)	0.70 (0.59-0.97)	0.73 (0.60-1.10)	0.59 (0.50-0.93)
UPCR^*^ (mg/g) (n=25)	1.05 (0.22-3.41)	1.94 (0.80-4.90)	0.19 (0.11-2.04)
Low C3 (n=29)	34%	42%	20%
Delivery (n=35)			
Maternal age^†^ (years)	26.9 ± 4.8	27.0 +/- 5.4	26.7 +/- 3.8
Cesarean section	46%	59%	23%
Gestational age^†^ (weeks)	33.8 ± 4.6	31.1 ± 3.8	38.2 ± 1.0
Birth weight^*^ (grams) (n=37)	2230 (1292–2583)	1532 (1046-2324)	2567 (2477-3147)

^*Median (interquartile range); †Mean ± standard deviation; UPCR, urine protein-to-creatinine ratio.^

First trimester data revealed potential LN disease activity as evidenced by a median urine protein-to-creatinine ratio (UPCR) of 0.86 mg/g and a low C3 level in 50% of pregnancies. These markers persisted into the third trimester, with a median UPCR of 1.05 mg/g and a low C3 level in 34% of pregnancies. The presence of a UPCR >0.5 mg/g in the first trimester was associated with preterm delivery (81% vs. 36%, *p* = 0.04). Other first trimester clinical markers, such as low C3, hypertension, and serum creatinine >0.8 mg/dl, were not significantly associated with preterm delivery. Hydroxychloroquine use was present in 77% in the first trimester and in 83% at delivery discharge. The use of azathioprine in the first trimester, but not hydroxychloroquine or prednisone, was associated with significantly less preterm birth (25% vs. 70%, *p* = 0.04). Calcineurin inhibitors (CNIs) were not administered in any pregnancy during the first trimester.

Preeclampsia (including superimposed and preeclampsia with severe features) was present in 40% (14/35) of all deliveries, HELLP syndrome in 5.7% (2/35), and preterm premature rupture of membranes in 17% (6/35). The median UPCR at the time of delivery was 3.5 g (IQR = 1.1–5.4, *n* = 30). All deliveries resulted in live births, although the neonatal outcomes (neonatal intensive care unit length of stay or subsequent neonatal death) were not followed up. Four individuals were on dialysis at the time of delivery: one had end-stage kidney disease at conception, two had chronic kidney disease (CKD) in the first trimester, and one had a new diagnosis of systemic lupus erythematosus (SLE) and class IV LN in the second trimester of pregnancy. The range of the gestational ages of infants born to individuals receiving dialysis was 25–34 weeks.

Among deliveries with preconception kidney biopsies (*n* = 24), the median duration of LN (measured from the date of the first kidney biopsy to the date of delivery) was 3.9 years. A duration of LN of 4 years or more was not associated with preterm delivery (36% vs. 62%, *p* = 0.4). Most deliveries with preconception biopsies occurred following the first kidney biopsy (15/24, 63%); nine (38%) occurred following two or more kidney biopsies. Overall, a pregnancy occurring within 2 years after a kidney biopsy was more likely to result in preterm birth than if the biopsy was performed more than 2 years prior to conception (82% vs. 23%, *p* = 0.01). Initial kidney biopsy after delivery took place in 26% (9/35) at a median of 5.4 months (IQR = 1.7–10.9) postpartum. Most postpartum biopsies (89%) occurred following a preterm delivery. At delivery, five of the nine individuals had a serum creatinine of 0.8 mg/dl or higher, and eight of the nine individuals had a UPCR of 3 g/g or higher.

Half of all the kidney biopsies held a primary diagnosis of class IV LN, while 26% were classified as a combined lesion (i.e., class III and V or class IV and V, *n* = 9). Among all biopsies, class IV LN (isolated or in combination with class V) was not significantly associated with preterm delivery (see **Table 2**). Preterm birth appeared to be more common among those with glomerular crescents (89% among those with >20% crescents on biopsy vs. 50% of those with <20% crescents, *p* = 0.06), although this did not reach statistical significance. Increased amounts of activity, chronicity, and interstitial sclerosis on biopsy were not significantly associated with preterm birth (**Table 2**).

**Table 2:** Comparison of biopsy characteristics and preterm birth (< 37 weeks’ gestation).

Variable	Preterm birth n (%)	Full-term birth n (%)	P value
Activity index > 6 (n = 15/24)	10 (67%)	5 (33%)	1.0
Chronicity index > 3 (n = 12/26)	10 (83%)	2 (17%)	0.22
Crescents > 20% (n = 9/33)	8 (89%)	1 (11%)	0.06
More than mild interstitial sclerosis (n = 6/32)	5 (83%)	1 (17%)	0.37
Primary Diagnosis Class IV LN (n = 17/35)	13 (76%)	4 (24%)	0.16
Primary Diagnosis Proliferative LN (n = 25/35)	17 (68%)	10 (40%)	0.44
Proliferative and membranous LN (n = 9/35)	5 (56%)	4 (44%)	0.70
Isolated Membranous LN (n = 8/35)	4 (50%)	4 (50%)	0.43

## Discussion

In this retrospective cohort, we found that preterm delivery was common in pregnant patients with LN, occurring in 63% of deliveries compared with 9.7%–10.7% of deliveries across the state of North Carolina during a similar time frame (11). In addition, those with LN who had first trimester proteinuria >0.5 g/g or underwent a kidney biopsy within 2 years of conception were more likely to experience preterm delivery. Specific biopsy findings of crescentic disease also emerged as a potential risk marker, although other specific histologic features, such as proliferative lesions and an increased activity index, did not. However, our small sample size and the incomplete histologic data limited the power of our analyses.

Other studies examining the LN class and maternal–fetal outcomes have shown mixed results. Among a retrospective cohort of 66 pregnancies in Brazil, higher rates of preeclampsia occurred with proliferative LN vs. class II or V LN (31.5% vs. 8.3%, *p* = 0.05) (3). Bramham et al. did not find an association between histologic class and maternal outcomes, including preeclampsia and preterm birth, among 43 pregnancies with previous LN (1). Similar results were found in an Italian cohort, although the authors found that a longer duration of LN and a history of flares predicted later preeclampsia (2). From our preconception biopsies, a longer duration of LN was not significantly associated with preterm birth, possibly due to sustained remission in the years following the initial biopsy. Alternatively, those with an overall milder disease course may be more likely to conceive and/or pursue pregnancy years after diagnosis than those with active and progressive disease. We also did not find that individuals with more than one kidney biopsy prior to pregnancy (as a possible marker for relapsing disease) were at higher risk of preterm birth (data not shown). Notably, the clinical indication for biopsy was unknown; therefore, it is conceivable that some were performed for surveillance rather than relapsing disease. However, given the date range of our dataset and the clinical practice of UNC at that time, we believe that the majority were performed for concern of flares. More specific biopsy characteristics, such as crescents or the impact of combined histologic lesions, have not been reported in other studies.

Our study found that first trimester azathioprine was associated with significantly less preterm birth, which could reflect optimized preconception care. However, we recognize the limitations of electronic record abstraction on medication adherence. CNIs are currently incorporated into a multi-targeted approach to LN induction and maintenance therapy (12). While cyclosporine and tacrolimus are compatible with pregnancy (13), there are insufficient data regarding voclosporin in pregnancy, and it is currently recommended to avoid its use due to its alcohol content. In small numbers, tacrolimus has been found to be beneficial in the treatment of LN flares and for maintenance of remission in pregnancy (14). While CNIs were not utilized in the first trimester here, further research is warranted to assess their antiproteinuric and steroid-sparing qualities with pregnancy outcomes in LN.

The limitations of this study were its sample size and its retrospective design, which included deliveries occurring over a time span during advancements in maternal–fetal care. We included only those enrolled in our histopathologic database who delivered at our tertiary hospital, which likely biased our sample toward more medically complex individuals. In addition, the disease activity during pregnancy was difficult to assess. The median level of proteinuria was notably above the goal for remission in both the first and third trimesters, but distinguishing between active disease vs. chronic glomerulosclerosis and/or gestational proteinuria was not possible. A low C3 level was also common in the first trimester. Outside of pregnancy, data from repeat kidney biopsies have shown substantial discordance between the clinical and histologic markers of disease remission (15). The preconception C3 level has been found to be a predictor of preterm birth in pregnancies affected by SLE (16), but we did not find statistical significance in our analysis. More large-scale, multi-site, prospective studies are needed for individuals with LN who are planning pregnancy in order to gain a more comprehensive understanding of preconception disease activity, treatment and response, and the impact these have on pregnancy outcomes, including preterm birth and preeclampsia.

We chose to use preterm birth as our primary outcome given the lack of a consensus diagnosis for preeclampsia in those with underlying hypertension and proteinuria. Patients with SLE have a higher risk of preeclampsia compared to those without (odds ratio = 3.0) (17, 18). International studies have also shown that LN carries a higher risk of preeclampsia than SLE alone (25.7% vs. 2.9% in a Swedish cohort and 28% vs. 16% in the UK) (1, 19). In the United States, patients with active LN (*n* = 23) compared with SLE patients without renal involvement (*n* = 47) were associated with a higher incidence of preeclampsia (26% vs. 11%) and were more likely to deliver preterm (median of 34 vs. 40 gestational weeks). In our cohort, the diagnosis of preeclampsia was made clinically and was present on the obstetric discharge summary or via delivery ICD codes. The patients with LN in our cohort were at high risk of preeclampsia, with risk factors that included chronic kidney disease, hypertension, and proteinuria. We did not have data on the *in vitro* fertilization rates that may have also increased risk. Acknowledging that race is a social construct, over half of our cohort was classified as African American, which has been associated with a 45% increased odds of preeclampsia (20). In our cohort, the high rates of probable disease activity in pregnancy (as evidenced by the first trimester findings of low C3 and proteinuria), could have also contributed to the high rate of preeclampsia. Furthermore, the preeclampsia diagnosis could have been misclassified based on the presence of chronic hypertension and proteinuria related to underlying LN. Our outcome of preterm delivery incorporated etiologies from preterm premature rupture of membranes, found in 17% of our deliveries, and the induction of labor to prevent superimposed preeclampsia, which are known causes of preterm delivery in those with SLE (1, 21).

Our findings add to the growing research regarding maternal–fetal health and LN. Consistent with prior studies, we found that preterm birth is common in pregnancies complicated by LN and that first trimester proteinuria increases the risk of preterm delivery. The association of the time from the most recent biopsy with preterm birth is a novel finding. We hypothesize that this may be a surrogate for disease activity and that a 2-year delay from diagnostic kidney biopsy could allow sufficient time to achieve disease remission, reducing the risk of preterm birth. Overall, these data could aid family planning discussions and promote preconception disease optimization for patients and their providers. In addition, while crescentic LN may pose higher risks of preterm birth, larger multi-site studies are necessary to assess how histologic features negatively impact obstetric outcomes.

## Data availability statement

The raw data supporting the conclusions of this article will be made available by the authors, without undue reservation.

## Ethics statement

This study was reviewed and approved by the Institutional Review Board of UNC (study number 20-1214). The studies were conducted in accordance with the local legislation and institutional requirements. Written informed consent for participation was not required from the participants or the participants’ legal guardians/next of kin in accordance with the national legislation and institutional requirements.

## Author contributions

MR: Conceptualization, Formal analysis, Investigation, Writing – original draft, Writing – review & editing. KG: Writing – review & editing. TM: Writing – review & editing. CP: Data curation, Writing – review & editing. LB: Data curation, Writing – review & editing. AS: Writing – review & editing. SH: Methodology, Supervision, Writing – review & editing. RF: Supervision, Writing – review & editing. VD: Conceptualization, Methodology, Supervision, Writing – review & editing.
